# *KDM2B*‐associated paunch calf syndrome in Marchigiana cattle

**DOI:** 10.1111/jvim.15789

**Published:** 2020-06-09

**Authors:** Leonardo Murgiano, Gianfranco Militerno, Fiorella Sbarra, Cord Drögemüller, Joana G. P. Jacinto, Arcangelo Gentile, Marilena Bolcato

**Affiliations:** ^1^ Department of Clinical Sciences & Advanced Medicine University of Pennsylvania Philadelphia Pennsylvania USA; ^2^ Department of Veterinary Medical Sciences University of Bologna Bologna Italy; ^3^ National Association of Italian Beef‐Cattle Breeders Perugia Italy; ^4^ Institute of Genetics, Vetsuisse Faculty, University of Bern Bern Switzerland

**Keywords:** bovine, genetic diseases, introgression, PCS

## Abstract

**Background:**

Chianina, Romagnola, and Marchigiana are the 3 most important Italian breeds of cattle raised in the Apennine Mountains. Inherited disorders have been reported in the Chianina and Romagnola breeds but not in the Marchigiana breed. Recently, a case resembling recessively inherited *KDM2B*‐associated paunch calf syndrome (PCS) in Romagnola cattle was identified in Marchigiana cattle.

**Hypothesis/Objectives:**

To characterize the features of the observed congenital anomaly, evaluate its possible genetic etiology, and determine the prevalence of the deleterious allele in the Marchigiana population.

**Animals:**

A single stillborn Marchigiana calf was referred for clinicopathological examination because of the presence of PCS‐like morphological lesions.

**Methods:**

The animal was necropsied and the calf and its parents were genotyped. A PCR‐based direct gene test was applied to determine the *KDM2B* genotype and 114 Marchigiana bulls were genotyped.

**Results:**

The pathological phenotype included facial deformities, enlarged fluid‐filled abdomen, and hepatic fibrosis. The affected animal was the offspring of consanguineous mating and homozygous presence of the *KDM2B* missense variant was confirmed. Both parents were heterozygous for *KDM2B* and the prevalence of carriers in a selected population of Marchigiana bulls was <2%.

**Conclusions and Clinical Importance:**

The characteristic malformations and genetic findings were consistent with the diagnosis of PCS and provide evidence that the deleterious *KDM2B* variant initially detected in Romagnola cattle also occurs in the Marchigiana breed.

**Abbreviations**
AI: artificial insemination
OMIA: Online Mendelian Inheritance in Animals
PCS: paunch calf syndrome
PMT: congenital pseudomyotonia

## 1 INTRODUCTION

Chianina, Marchigiana, and Romagnola are the 3 most common Italian cattle breeds in the central part of the Apennine Mountains. Although used in the past mainly as draft animals, in recent decades they have been included in intensive selective breeding programs to increase their use for beef production.^1^, ^2^, ^3^ While pursuing improvement in the performance traits, it also is important to limit the undesirable collateral effects of inbreeding because the accumulation of harmful alleles may lead to emergence of recessively inherited disorders. Many such disorders have been cataloged in the Online Mendelian Inheritance in Animals (OMIA) database,^4^ and identification of recessive pathogenic variants allows targeted genotyping and the avoidance of high‐risk matings of carrier animals.^5^ Although many are unknown and routine screenings are not performed, recessive variants can quickly spread through a population by widespread use of popular carrier bulls in artificial insemination (AI). This practice led to the appearance of worrisome pathogenic variants in the Chianina and Romagnola breeds.^6^, ^7^, ^8^, ^9^, ^10^

In Chianina cattle, the c.491G>A missense variant in the *ATP2A1* gene, encoding the SERCA1 pump, was reported to cause autosomal recessive pseudomyotonia (PMT), a congenital muscle function disorder (OMIA 001464‐9913).^9^ Among the ranked Chianina sires in the years 2007 to 2011, the prevalence of heterozygous PMT carriers was 13.6%, illustrating the scale of the problem.^11^ Subsequently, *ATP2A1*‐associated PMT also was reported in the Romagnola breed associated with allelic heterogeneity of the *ATP2A1* gene.^10^ In addition to the previously reported c.491G>A missense variant originating from accidental introgression of a Chianina PMT carrier, 2 additional rare *ATP2A1* missense variants (c.632G>T and c.857G>T) also were found to be causative for the disorder.^10^

Ichthyosis fetalis caused by a missense variant in the *ABCA12* gene (harlequin ichthyosis; OMIA 002238‐9913) is another lethal recessively inherited disorder that occurs in the Chianina breed.^12^ A less severe form of congenital ichthyosis also was reported in Chianina cattle, but no causative genetic variant thus far has been found.^13^ More recently, rare recessively inherited congenital bilateral immature nuclear cataracts have been found in Romagnola cattle (OMIA 001936‐9913) and are associated with a large deletion affecting the coding region of the *NID1* gene.^14^ Previously, an outbreak of a lethal multiorgan developmental dysplasia was described in 65 Romagnola cattle (OMIA 001722‐9913) and determined to be caused by a c.2503G>A missense variant in the *KDM2B* gene.^6^, ^7^ On the basis of the phenotype, this disorder was named “paunch calf syndrome” (PCS). Affected calves usually are stillborn or die within hours of delivery.^6^, ^7^ The *KDM2B* gene encodes a histone demethylase that acts as an important transcription regulator affecting organ development and cell differentiation. The disease‐associated variant leads to an amino acid exchange in a highly conserved domain, thus explaining the phenotypic effect of the genetic variant.^7^ The PCS phenotype mainly is characterized by facial dysplasia, an enlarged and pendulous abdomen (“paunch”) with considerable abdominal effusion and hepatic fibrosis.^6^, ^7^ Additional lesions, such as cleft palate, lack of the medial dew claws of ≥1 limbs, SC edema, perihepatic cysts, and cardiac malformations are found in some cases.^6^, ^7^ Moreover, after identifying its molecular cause, results of a subsequent survey on the prevalence of carriers for PCS in the Romagnola breed were concerning. The prevalence of PCS carriers among top‐ranked Romagnola sires over the years 2007 to 2012 was 29.3%, and even higher (30.9%) among young males selected for performance testing.^15^

Recently, a Marchigiana calf with a complex congenital malformation phenotype resembling PCS of Romagnola cattle was presented for examination. Our aim was to describe the clinicopathological phenotype associated with the inherited disorder of *KDM2B* causing this disease entity in Marchigiana cattle.

## 2 MATERIALS AND METHODS

A full‐term stillborn, 55 kg, male Marchigiana calf delivered after dystocia was referred to the teaching hospital of the Department of Veterinary Medical Sciences of the University of Bologna for evaluation of multiple congenital malformations.

A complete phenotype study was performed. Representative samples from the liver were collected, fixed in 10% buffered formalin, embedded in paraffin, and processed for histological examination. Five‐micron histological sections of the liver were stained with hematoxylin and eosin, Masson's trichrome and rhodanine, and specific immunohistochemical methods, using the streptavidin‐biotin peroxidase technique, including vimentin (VIM; dilution 1:100; Dako, Glostrup, Denmark) and α‐smooth muscle actin (α‐SMA; dilution 1:100; Dako) were applied to formalin‐fixed liver samples.

Additionally, a pedigree analysis of the affected calf was performed. We harvested cutaneous tissue from the calf, as well as EDTA blood from its parents. Desoxyribonucleic acid was extracted and the 3 subjects were genotyped for the c.2503G>A variant in the *KDM2B* gene, as previously described^7^ and implicated in PCS in Romagnola cattle.^7^ Furthermore, 114 samples were collected for *KDM2B* genotyping, including semen samples from 87 Marchigiana adult bulls that were classified as suitable for reproduction and an additional 27 EDTA blood samples from young males selected for performance testing.

## 3 RESULTS

### 3.1 Clinical phenotype

**FIGURE 1:**
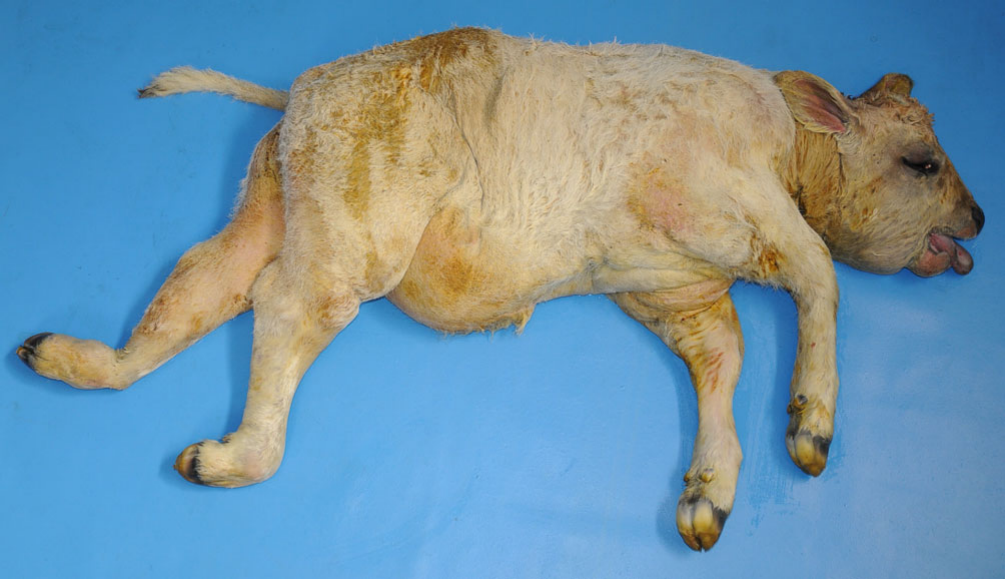
Stillborn Marchigiana calf showing multiple congenital anomalies. Note the shortened face and distended ventral abdomen resembling paunch calf syndrome reported in Romagnola cattle. Note the swollen hind legs and the right fetlock externally rotated

The affected calf had an enlarged head caused by a SC swelling that was especially evident in the periocular and the submandibular regions. The splanchnocranium was shortened and the frontal region asymmetric. The tongue was swollen and protruded from the mouth. The eyes were enlarged with evident scleral injection and conjunctival edema. The ventro‐abdominal region was enlarged and pendulous and generalized swelling was noticed. The hind legs also were swollen and the right fetlock was externally rotated (Figure 1).

### 3.2 Pathological phenotype

The swellings were caused by generalized SC infiltration of serosanguineous fluid, as evidenced at necropsy. Similar fluid collection also was present in the peritoneal, pleural, and pericardial cavities.

The liver was larger than normal, with an irregular surface and firm consistency. Upon sectioning, the cut surface of the liver had numerous bands of whitish, collagenous connective tissue, and a lobular structure partitioned by fibrous septa, which also were visible inside the lobules. Two round 5‐mm diameter cysts, containing reddish fluid, were present on the peritoneal surface of the left lobe of the liver.

**FIGURE 2:**
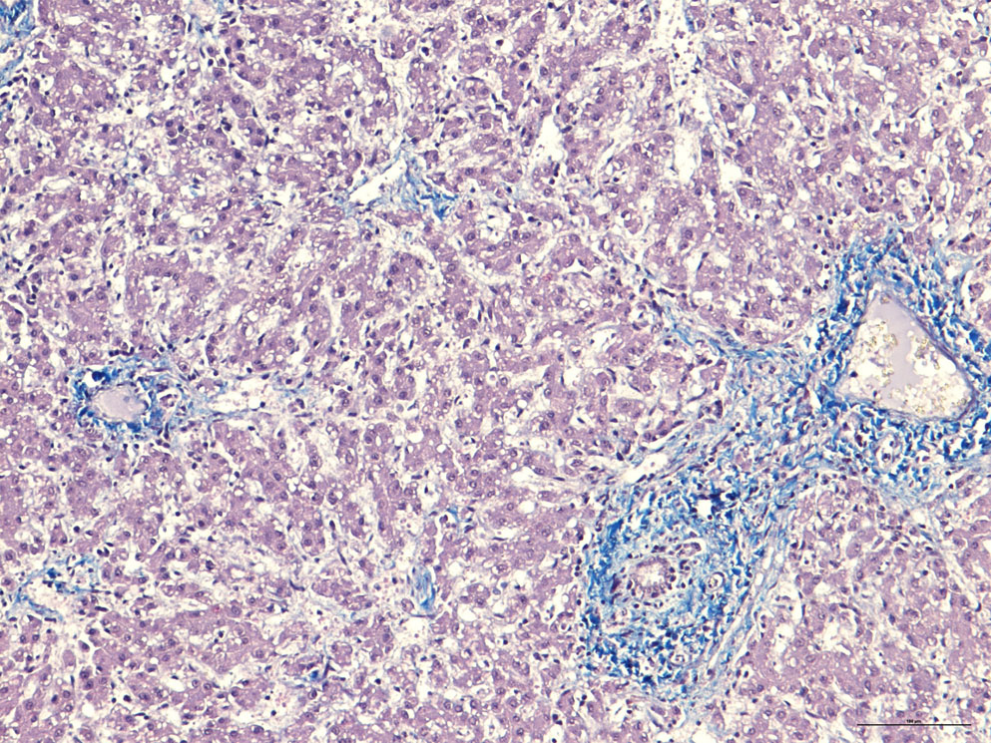
Histological section from the liver of the stillborn Marchigiana calf. Note the slight to moderate fibrosis in the portal fields and, in some cases, around the central space of the liver lobule. The fibrosis, characterized by the formation of thin portal‐portal and portal‐central fibrotic septa, extends, in some lobules, to the perisinusoidal spaces. There are many enlarged sinusoids and cellular hepatic degeneration, possibly due to autolysis phenomena. Masson's trichrome, bar = 100 μm

Histopathology identified extensive distortion of the lobular architecture by slight to moderate fibrosis in the portal fields and, in some cases, around the central space of the liver lobule (hematoxylin and eosin staining). The fibrosis was more evident in sections stained with Masson's trichrome, and was characterized by the formation of thin portal‐portal and portal‐central fibrotic septa. In some lobules, fibrosis extended to the perisinusoidal spaces. Fusiform cells were observed in the inner wall of the sinusoids. Enlarged sinusoids, cellular hepatic degeneration or atrophy, and capsular fibrotic thickening were detected. No copper deposits responsible for fibrogenesis were detected on rhodanine staining. Immunohistochemical examination (vimentin and α‐smooth muscle actin) identified slight immunoreactivity for laminin intermediate filaments. The fusiform cells, positive using antivimentin and anti‐α‐smooth muscle actin antibodies and observed within the sinusoids in fibrotic areas, were considered myofibroblasts actively involved in fibrogenesis (Figure 2). Histopathologically, cysts had an inner wall lined by a single layer of cuboidal biliary epithelium and were considered to represent bile duct dilatation associated with fibrosis.

### 3.3 Genetic analysis

**FIGURE 3:**
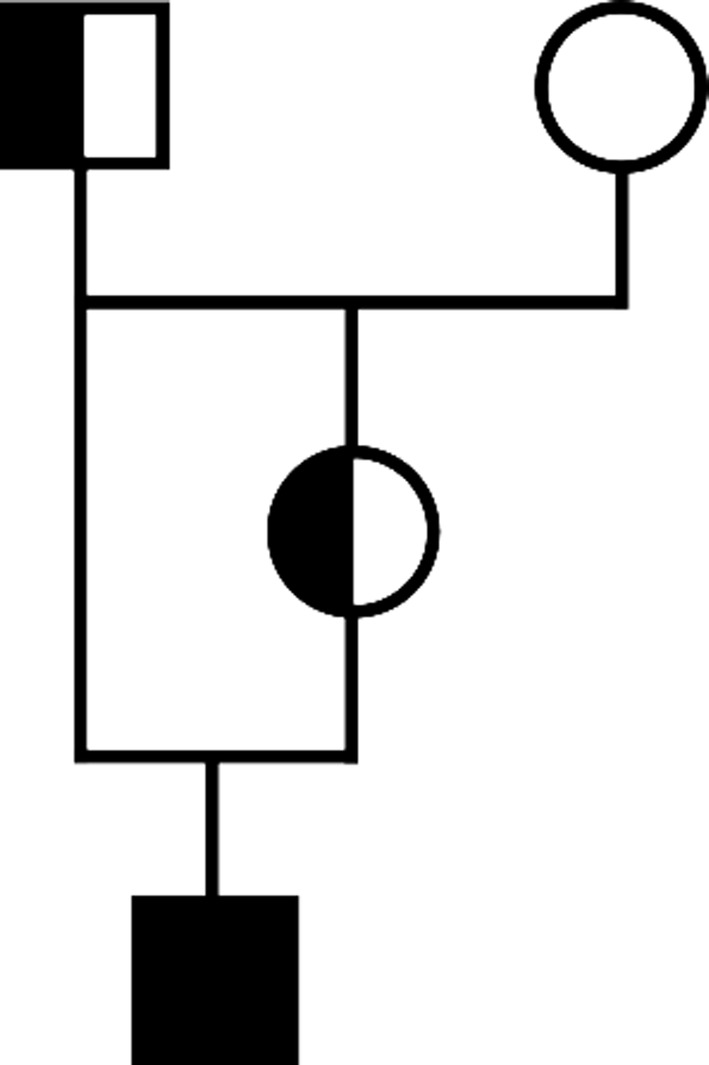
Pedigree of the stillborn Marchigiana calf. Note the inbreeding loop and the homozygous genotype of the case related to the pathogenic *KDM2B* variant (black symbol) and the half‐filled symbols showing the parents as carriers

Pedigree analysis of the stillborn Marchigiana calf disclosed the presence of a consanguineous mating, because the sire of the affected calf was also the sire of its dam (Figure 3). In light of this inbreeding, autosomal recessive inheritance seemed a likely explanation for the occurrence of the congenital anomaly. The homozygous presence of the *KDM2B* variant subsequently was confirmed in the affected animal, and both parents were confirmed as heterozygous carriers of this deleterious allele. Evaluation of the prevalence of this pathogenic variant in Marchigiana cattle identified a frequency of 1.75% (2 of the 114 tested bulls were heterozygous carriers).

## 4 DISCUSSION

The clinical and pathological findings described in the calf of our study completely overlapped with the phenotype reported for PCS of the Romagnola breed. Therefore, our report constitutes the first description of a *KDM2B* homozygous mutant case of PCS in Marchigiana cattle, confirming an identical genetic etiology for a disorder that has been known to exist for many years in Romagnola cattle. It is possible that the origin of this rare disease‐causing *KDM2B* variant in Marchigiana breed is based on either accidental crossbreeding or targeted introgression of Romagnola cattle. In the available pedigree data for the presented case, no indication of cattle other than the Marchigiana breed was found. Present day Marchigiana cattle are derived from Podolian cattle, a stock typical of the Italian Marche region that originated in the region of Podolia in present day Ukraine. The breed was introduced to Italy after the fall of the Roman Empire and in the past was used for draft work. In the late 19th and early 20th centuries, the Marchigiana breed was improved first by crossing with the Chianina breed and successively by further crossing with the Romagnola breed (also deriving from Podolian cattle). Since the 1930s, the breed has undergone appropriate breeding strategy that has directed it toward beef production, resulting in 1 of the most competitive beef breeds in Italy that also is exported throughout the world.^1^

Unfortunately, no information is available to determine when the PCS‐associated *KDM2B* variant was introduced into the Marchigiana breed. In fact, it cannot be excluded that the variant already was present before the Marchigiana breed was established as distinct breed, and therefore it may represent the identical variant as in the Romagnola breed. To the best of our knowledge, no other cases have been reported, and it is possible that the frequency of the deleterious allele is very low.

This speculation was confirmed by the genotyping of 114 bulls used for AI. Nevertheless, additional data might be useful to make a more precise estimate. For this purpose, DNA isolated from skin biopsy samples or semen should be used to determine the individual's genotype without concern about cattle‐specific leukochimerism possibly causing a false genotype.^16^ The occurrence of the PCS‐causing variant in another historically related breed is similar to what we have observed before in PMT‐affected Romagnola cattle, carrying the same disease‐causing allele previously detected in the Chianina breed.^10^

Compared to the substantial allele frequencies of the PCS variants in Romagnola cattle (14.6% in top‐ranked sires and 15.4% in young bull calves),^15^ the low prevalence (<2%) suggests that the variant is not widespread within the Marchigiana population. The observed phenotype in the affected stillborn Marchigiana calf differs somewhat from the PCS cases in Romagnola cattle reported previously,^6^, ^7^ specially the swollen hind limbs and rotated right fetlock. Nonetheless, the most striking features of the disorder such as the short face, pendulous abdomen, serosanguineous fluid in the abdominal cavity, and hepatic fibrosis strongly resemble the previously described PCS phenotype. The observed differences may have reflected the different genetic background of Marchigiana breed.

Our report serves to alert breeders of Marchigiana cattle about the possible emergence of PCS in the future and will permit the avoidance of carrier matings by systematic genetic testing of potential sires.

## CONFLICTS OF INTERESTS DECLARATION

Authors declare no conflict of interests.

## OFF‐LABEL ANTIMICROBIAL DECLARATION

Authors declare no off‐label use of antimicrobials.

## INSTITUTIONAL ANIMAL CARE AND USE COMMITTEE (IACUC) OR OTHER APPROVAL DECLARATION

This study was not based on an invasive animal experiment and used a naturally occurring case, therefore there are no associated permit numbers. The blood used for the genetic analysis derives from samples obtained for sanitary controls or for other reasons not related to this investigation. The investigation cannot be considered including “animal experiment” according to the exemptions contemplated by the Italian legislative decree n. 26/2014 (Dir. 2010/63/UE on the protection of animals used for scientific purposes).

## HUMAN ETHICS APPROVAL DECLARATION

Authors declare human ethics approval was not needed for this study.
